# Engineering 3D Printed Scaffolds with Tunable Hydroxyapatite

**DOI:** 10.3390/jfb13020034

**Published:** 2022-03-23

**Authors:** Yoontae Kim, Eun-Jin Lee, Anthony P. Kotula, Shozo Takagi, Laurence Chow, Stella Alimperti

**Affiliations:** 1 American Dental Association Science & Research Institute, Gaithersburg, MD 20899, USA; kimyo@ada.org (Y.K.); leee@ada.org (E.-J.L.); takagis@ada.org (S.T.); chowl@ada.org (L.C.); 2 Materials Science and Engineering Division, National Institute of Standards and Technology, Gaithersburg, MD 20899, USA; anthony.kotula@nist.gov

**Keywords:** 3D printing, osteoclast, HA, CPC, TTCP, DCPA, tunable material

## Abstract

Orthopedic and craniofacial surgical procedures require the reconstruction of bone defects caused by trauma, diseases, and tumor resection. Successful bone restoration entails the development and use of bone grafts with structural, functional, and biological features similar to native tissues. Herein, we developed three-dimensional (3D) printed fine-tuned hydroxyapatite (HA) biomimetic bone structures, which can be applied as grafts, by using calcium phosphate cement (CPC) bioink, which is composed of tetracalcium phosphate (TTCP), dicalcium phosphate anhydrous (DCPA), and a liquid [Polyvinyl butyral (PVB) dissolved in ethanol (EtOH)]. The ink was ejected through a high-resolution syringe nozzle (210 µm) at room temperature into three different concentrations (0.01, 0.1, and 0.5) mol/L of the aqueous sodium phosphate dibasic (Na_2_HPO_4_) bath that serves as a hardening accelerator for HA formation. Raman spectrometer, X-ray diffraction (XRD), and scanning electron microscopy (SEM) demonstrated the real-time HA formation in (0.01, 0.1, and 0.5) mol/L Na_2_HPO_4_ baths. Under those conditions_,_ HA was formed at different amounts, which tuned the scaffolds’ mechanical properties, porosity, and osteoclast activity. Overall, this method may pave the way to engineer 3D bone scaffolds with controlled HA composition and pre-defined properties, which will enhance graft-host integration in various anatomic locations.

## 1. Introduction

Bone defects caused by trauma, dental diseases, and surgical tumor resection require reconstruction to restore bone structure and function [1,2,3]. Current options to treat these defects include the use of autografts which may be inadequate by supply and demonstrate limitations due to high morbidity. For these reasons, allografts have been introduced and tested as an alternative treatment option. However, they show limitations associated with lower host incorporating properties than autografts. In addition, they develop immune reactions and may transmit infectious agents, even if they are procured, processed, and distributed only by tissue banks, which operate under strict guidelines and sterile conditions to minimize the abovementioned issues [4,5]. Finally, commercial products containing bone tissue from different animal sources, named xenografts, have been utilized. Although they are abundant in supply, they have limited success because of the risks of transmission of zoonotic diseases and the elimination of the organic components to render the material nonimmunogenic, which diminishes the material properties and the graft incorporation [6,7,8]. Thus, new bone engineering strategies may help produce constructs that closely mimic structural, functional, and biological features to properly incorporate the graft into the host tissue and restore bone function [9,10,11,12]. An essential process for bone restoration and graft incorporation is bone resorption. The primary mechanism of bone graft remodeling is initiated by hematoma formation around the implanted graft, followed by an inflammatory response and the formation of the fibrovascular stroma. Next, osteogenic precursor cells infiltrate the graft, and new bone formation is initiated. In addition, the graft resorption was started by multinuclear cells and osteoclasts [2]. The resorption rate needs to be similar to the rate of new bone formation for successful graft remodeling [13,14].

Scaffolds, fabricated by traditional methods such as gas foaming [15,16], freeze-drying [17,18], leaching [19], and emulsification [20,21], cannot control the pore shape, architecture, porosity, or interconnectivity of the scaffolds and thus cannot precisely and adequately improve cell growth, osteogenesis, and resorption. The development of 3D printing using computer-aided design (CAD) and computer-aided manufacturing (CAM) has significantly advanced the fabrication of customized scaffolds in a patient-specific manner with tunable chemistry, controlled mechanical properties, designed shapes, and interconnected porosity [22,23,24]. Materials such as bioceramic powders, including hydroxyapatite (HA), β-tricalcium phosphate (β-TCP), carbonate calcium deficient hydroxyapatite (CDHA) combined with biodegradable and biocompatible polymers such as poly (l-lactic acid) (PLLA), poly(vinyl alcohol) (PVA), polycaprolactone (PCL), and natural or tunable hydrogels have been adopted as inks for extrusion-based printing [25,26,27,28,29,30]. The printing process to engineer the extrusion-based 3D printed scaffolds requires the assistance of high-temperature melting-extrusion, sintering, or further stabilization via ultraviolet (UV)-, ion-, or temperature-assisted crosslinking [22,23,24]. Moreover, they demonstrate limited printing resolution and only mimic the hierarchical heterogeneous structure of bone at a relatively low level with inadequate mechanical properties [22,23,24]. Thus, new 3D printing strategies need to be developed to enable the fabrication of 3D printed bone scaffolds with controlled structural and mechanical properties.

In this study, we fabricated high-resolution 3D printed bone scaffolds with controlled HA composition amount, porosity, and tunable mechanical properties. The 3D printed scaffolds were formulated from non-aqueous calcium phosphate cement (CPC) precursors containing tetracalcium phosphate (TTCP; Ca_4_(PO_4_)_2_O) and dicalcium phosphate anhydrous (DCPA; CaHPO_4_) [1]. The slurry was extruded through a 210 μm nozzle into a sodium phosphate dibasic (Na_2_HPO_4_) bath at three different concentrations (0.01 mol/L, 0.1 mol/L, and 0.5 mol/L). Our results showed that the different concentrations of Na_2_HPO_4_ affected the reaction rate of HA formation and the total amount of the formed in situ HA. In addition, the concentration of the aqueous solution contributed to the scaffold’s porosity, mechanical properties, and resorption. Overall, these 3D printed tunable HA scaffolds with defined mechanical, structural, and porous properties will enable us to apply them as bone grafts in various anatomic locations, such as oral and craniofacial areas.

## 2. Materials and Methods

### 2.1. CPC Ink and 3D Printing Method

**Figure 1:**
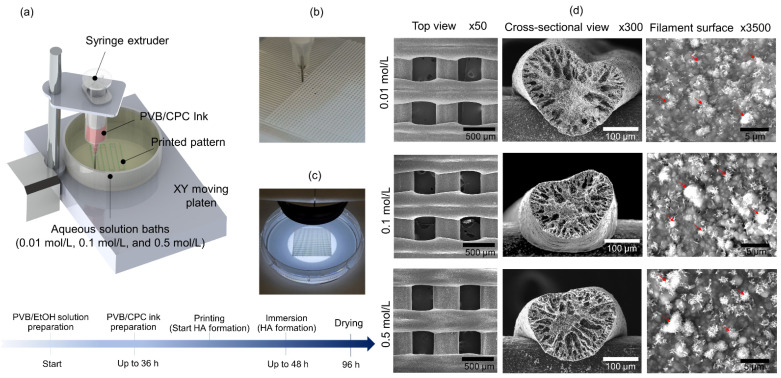
Fabrication of the 3D printed HA tunable scaffolds. (**a**) Schematic drawing of the 3D printing process to produce the Polyvinyl butyral (PVB)/Hydroxyapatite (HA) scaffolds in the presence of 0.01 mol/L, 0.1 mol/L, and 0.5 mol/L Na_2_HPO_4_; (**b**) Representative image of the 3D printed in situ HA scaffolds using 210 µm diameter nozzle; (**c**) Representative image of the 3D printed scaffolds (2 layers) in the aqueous solution (0.1 mol/L); (**d**) SEM images of Top view (Scale Bar = 500 μm), Cross-sectional view (Scale Bar = 100 μm), and Filament surface (Scale Bar = 5 μm) of the dried scaffolds after immersed 0.01 mol/L, 0.1 mol/L, and 0.5 mol/L Na_2_HPO_4_ solutions.

The CPC powder (73% (by a mass fraction) of the TTCP (Ca_4_(PO_4_)_2_O) and 27% (by a mass fraction) of the DCPA (CaHPO_4_)) was added to the PVB/EtOH solution (25 g per 100 mL EtOH) at a mass ratio of 0.75 to 1 [1]. The slurry was filled into the syringe and ejected through a 210 µm (27 gauge) nozzle of a 3D bioprinter (Rokit Healthcare, INVIVO) into three concentrations of Na_2_HPO_4_ solutions (0.01 mol/L, 0.1 mol/L, and 0.5 mol/L). Next, the 3D printed scaffolds were placed into Na_2_HPO_4_ solutions (0.01 mol/L, 0.1 mol/L, and 0.5 mol/L) for 48 h and then dried at 25 *°*C for 48 h (Figure 1a–c). The specific printing parameters are shown in Table S1.

### 2.2. Long-Term Raman Analysis

The 3D printed scaffolds were embedded in three different concentrations (0.01 mol/L, 0.1 mol/L, and 0.5 mol/L) of Na_2_HPO_4_ solutions. They were placed into a glass-bottom container and analyzed for 48 h using a rheo-Raman microscope, consisting of a Raman spectrometer (DXR Raman Microscope; Thermo Scientific; Waltham; USA) coupled to a rheometer (HAAKE MARS III; Thermo Scientific) [31]. The rheometer function was not used for this work. The laser (780 nm) was focused on the center of the filaments using a microscope objective (LMPLFLN10×; Olympus; Waltham, MA, USA), and spectra were measured using a high-resolution grating, 830 grooves per mm. The total spectral range was between 500 cm^−1^ and 1500 cm^−1^. Spectra were collected with a 10 s exposure time, and iterative Raman spectra measurements were automatically generated using the Macros Basic software for OMNIC.

### 2.3. XRD Analysis

The formation of HA in the scaffolds was obtained through X-ray powder diffraction (XRD) (Rigaku; SmartLab; Spring; USA). The XRD θ/2θ scans were collected on the 3D printed scaffolds with dimensions of 20 mm (length) × 7 mm (width) × 1 mm (height) at room-temperature with Cu Kα radiation generated at 40 kV and 44 mV. The scanning range was from 20° to 50° with 0.01° step and the 2°/min in speed.

### 2.4. Scanning Electron Microscopy

The topography and morphology of the 3D printed scaffolds, which were vertically cut, mounted on aluminum sample stubs, and coated with a thin film sputter (Desk V; Denton Vacuum; Moorestown, NJ, USA), were examined by JSM-IT1500SEM (JEOL; Peabody; USA). The accelerating voltage and the working distance were set to 5 kV and 8 mm, respectively.

### 2.5. Mechanical Properties Measurement

The 3D printed sheets with 100% infill rate were prepared in the dimension of 23 mm (length) × 5 mm (width) × 0.5 mm (height) and were mounted on the sample holder. 100 N load was applied at the center of the specimens by using a three-point bending machine (Model 1122, InstruMet; Union; USA).

### 2.6. Microcomputed Tomography (µCT) Measurements

The printed filament samples were inserted in the sample holder (U40830), which was attached to a rotational stage of μCT (Model µCT 40, SCANCO Medical AG; Wangen-Brüttisellen, Switzerland). The X-ray parameters were 70 kV and 140 µA for 690 images. The porosity was calculated through post-processing in the µCT evaluation program V6.5 (SCANCO Medical AG; Wangen-Brüttisellen, Switzerland).

### 2.7. Raw 264.7 Culture and Differentiation

The 3D printed scaffolds with dimensions (3 mm (length) × 3 mm (width) × 0.5 mm (height)) were inserted in 48-well plate and sterilized with 70% ethanol (volume fraction in water) and treated with ultraviolet (UV) irradiation for 12 h. The murine RAW 264.7 cell line (ATCC; Manassas, VA, USA) were plated at 0.5 million cells/mL density on each scaffold, and after 24 h, the cells were attached and cultured in Growth media (Dulbecco modified Eagle’s medium (Gibco), supplemented with 10% (by volume fraction) fetal bovine serum (Thermo Scientific; Waltham, MA, USA), 100 U/mL penicillin, and 100 µg/mL streptomycin) for 3 d_._ Next, to induce the differentiation of the cells toward osteoclasts, the cells were cultured in Differentiation media (Dulbecco modified Eagle’s medium (Thermo Scientific; Waltham; USA), supplemented with 10% (by volume fraction) fetal bovine serum (Thermo Scientific; Waltham, MA, USA), 100 U/mL penicillin, and 100 µg/mL streptomycin, and 50 ng/mL RANKL (Thermo Scientific; Waltham, MA, USA) for 7 d.

### 2.8. Immunostaining

Raw 264.7 lying on the 3D printed scaffolds were fixed in 4 g paraformaldehyde (Sigma; St. Louis, MI, USA) per 100 mL PBS for 20 min. They were permeated with 0.1% (by volume fraction) Triton X-100 in phosphate-buffered saline (PBS) for 20 min at room temperature, and then blocked with 0.01% (by volume fraction) Triton X-100 in PBS, 5 g/100 mL goat serum (Sigma; St. Louis, MI, USA) in PBS overnight at 4 *°*C. Next, they were stained with Tartrate-Resistant Acid Phosphatase (TRAP) using an Acid Phosphatase, Leukocyte (TRAP) Kit (Sigma; St. Louis, MI, USA) according to the manufacturer’s instructions. Finally, they were stained for cell viability/cytotoxicity using a LIVE/DEAD™ Viability/Cytotoxicity Kit (ThermoFisher; Waltham, MA, USA). Imaging was performed using a confocal microscope, and the analysis was performed by ImageJ [32].

### 2.9. Real Time Quantitative PCR Analysis

Total RNA was extracted from RAW 264.7 cells cultured on the scaffolds using TRIzol Reagent (Invitrogen) following the manufacturer’s instructions. 0.5 μg of RNA was reverse-transcribed by using the SuperScript III First-Stand Synthesis system (ThermoFisher; Waltham, MA, USA). Real-time quantitative polymerase chain reaction (RT-qPCR) experiments were performed in ViiA 7 Real-Time PCR System (Applied Biosystems™; Waltham, MA, USA) by using PowerTrack SYBR Green Master Mix (Applied Biosystems™; Waltham, MA, USA) and primers (Table S2), according to the manufacturer’s instructions. The target genes’ relative expression was calculated by using the standard curve method for the target Cq values and the Cq value for 18S ribosomal RNA (18S rRNA).

### 2.10. Statistical Analysis

All information regarding the number of experimental repeats and sample sizes were included in figure legends. Statistical analyses were performed using Microsoft Excel. All data points on the graphs represent average values, and error bars depict the standard error of the mean (S.E.M.). Statistical analyses were determined by one-way ANOVA with Tukey’s post-hoc test. *p* < 0.05 was considered statistically significant.

## 3. Results

### 3.1. Morphological Study of PVB/HA Composite Scaffolds

To fabricate 3D printed HA tunable scaffolds, we used PVB/CPC slurry composed of a liquid phase (PVB/EtOH solution) and a solid phase (a mixture of TTCP and DCPA) [1], which ejected through a 210 µm (27 gauge) nozzle into three concentrations of Na_2_HPO_4_ solutions (0.01 mol/L, 0.1 mol/L, and 0.5 mol/L) (Figure 1a–c). SEM images from the top view, the cross-sectional view, and the filament surface of the 3D printed scaffolds demonstrated the existence of the mineral phase (Figure 1d).

### 3.2. Real-Time Analysis of HA Formation

**Figure 2:**
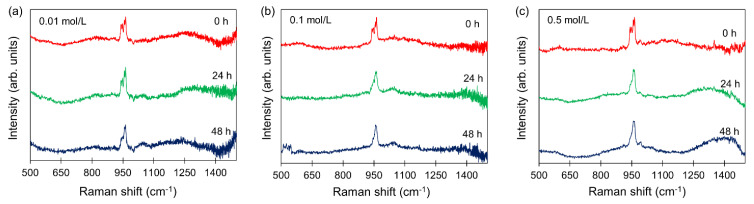
In situ real-time measurement of HA in the scaffolds. Long-term and real-time measurement of the HA by Raman spectroscopy under (**a**) 0.01 mol/L Na_2_HPO_4_; (**b**) 0.1 mol/L Na_2_HPO_4_; and (**c**) 0.5 mol/L Na_2_HPO_4_ solutions for 48 h.

Although SEM images demonstrated the presence of mineral phase in the scaffolds, they are limited to identifying the presence and crystal structure of HA. To this end, we performed Raman spectroscopy to capture the real-time in situ HA formation and TTCP reaction in the presence of 0.01 mol/L, 0.1 mol/L, and 0.5 mol/L Na_2_HPO_4_. The spectral of each material was measured as shown in Figure S1. Based on these spectral data of each material, we found that the peak centered at 960 cm^−1^ indicates the HA formation, and the peak centered at 940 cm^−1^ shows the presence of TTCP material (Figure S1). The TTCP peak was maintained at 940 cm^−1^ in the 3D printed scaffolds immersed in 0.01 mol/L aqueous solution for 48 h (Figure 2a). However, scaffolds immersed in 0.1 mol/L aqueous solutions and 0.5 mol/L aqueous solutions demonstrated a reduction in intensity at 940 cm^−1^ after 24 h. In addition, Raman intensity at 960 cm^−1^ was increased for both scaffolds formed in 0.1 mol/L and 0.5 mol/L aqueous solutions (Figure 2b,c). After 48 h soaking, the scaffolds using 0.1 mol/L and 0.5 mol/L solutions showed a similar spectral shape to commercially available HA c and Figure S1). Based on the measurement results, it is noted that the concentration of Na_2_HPO_4_ solution affected the speed of chemical reaction for HA formation.

**Figure 3:**
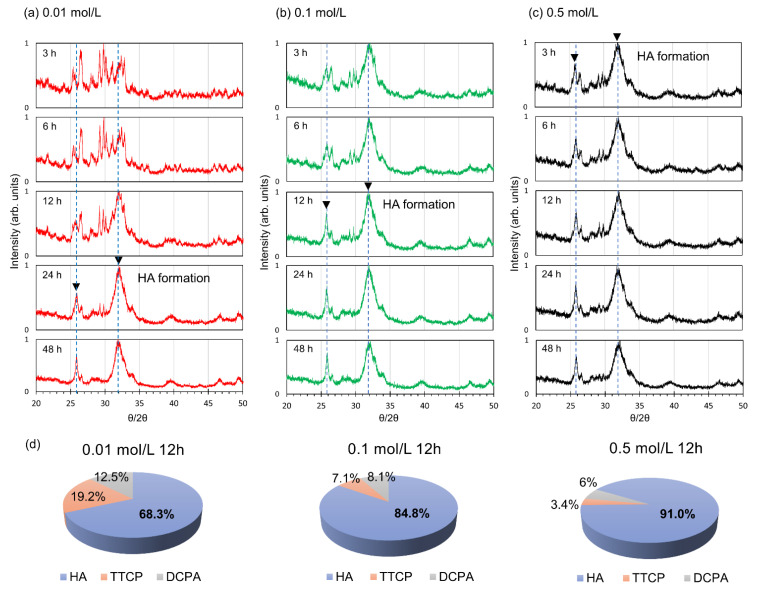
Effect of the printing environment on HA formation in 3D printed scaffolds. X-ray diffraction (XRD) scan of the 3D printed scaffolds embedded up to 48 h in (**a**) 0.01 mol/L Na_2_HPO_4_ solution; (**b**) 0.1 mol/L Na_2_HPO_4_ solution; (**c**) 0.5 mol/L of Na_2_HPO_4_ solution; (**d**) Representative data for 3D printed scaffold composition (in percent volume fraction) at 12 h time point by using whole powder pattern fitting (WPPF) method; *n* = 3 individual experiments.

In addition to Raman spectroscopy, we performed real-time analysis of HA by XRD. First, we examined each material’s standard θ/2θ values to identify the conspicuously displayed intensity peaks (Figure S2). The maximum intensity of HA was found at 31.8*°*, with peaks at 25.9*°*, 31.8*°*, 32.2*°*, and 32.9*°*. DCPA had peaks at 26.4*°*, 26.6*°*, and 30.2*°* (Max. intensity), while TTCP had peaks at 29.2*°* and 29.8*°* (Max. intensity) (Figure S2). We selected 25.9*°* and 31.8*°* (inverted black triangle) as the point at which HA formed on the scaffold based on this, and we examined the specimens immersed for 48 h at 0.01 mol/L, 0.1 mol/L, and 0.5 mol/L aqueous solutions. The source peaks of TTCP and DCPA were dominating at first when the scaffold was immersed in 0.01 mol/L aqueous solution, but after 24 h, strong HA peaks were visible at the two θ/2θ angles (25.9*°* and 31.8*°*) identified by us (Figure 3a). After 12 h, strong peaks were found from the scaffold immersed in 0.1 mol/L aqueous solutions (Figure 3b), and after 3 h, strong HA peaks were found from the scaffold immersed in 0.5 mol/L aqueous solutions (Figure 3c). Notably, higher Na_2_HPO_4_ concentration accelerates the formation of HA. In addition, we calculated the weight fraction of HA in scaffolds immersed in those aqueous solutions for 12 h by using the whole powder pattern fitting (WPPF) method, which calculates approximately the substances and fraction of the sample based on the measured XRD spectrum and the material database. Interestingly, our results showed that HA content was elevated as the Na_2_HPO_4_ concentration was increased. Specifically, the calculated content of HA was 68.3 ± 5% (volume fraction) in 0.01 mol/L aqueous solution, 84.8 ± 8% (volume fraction) in 0.1 mol/L aqueous solution, and 91.0 ± 2% (volume fraction) in 0.5 mol/L aqueous solutions, as shown in Figure 3d.

### 3.3. Structural and Mechanical Properties of the 3D Printed Scaffolds

**Figure 4:**
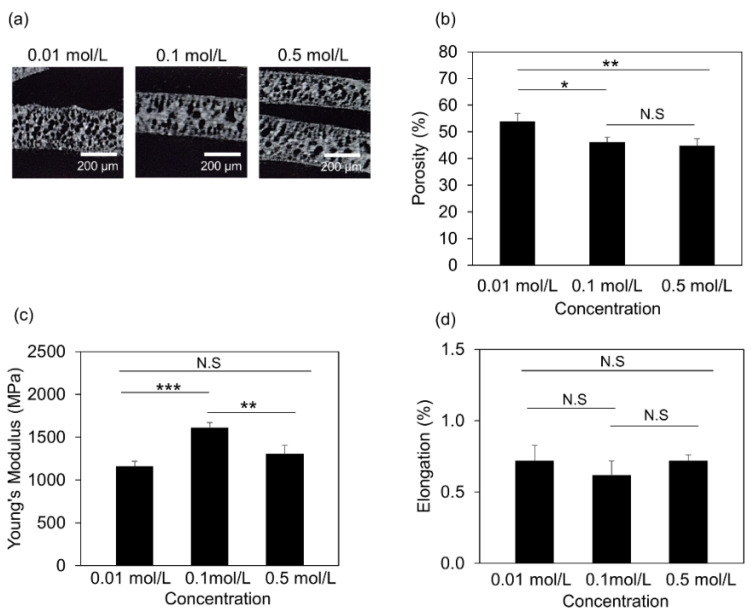
Microstructural analysis of 3D printed scaffolds: (**a**) Representative μCT images of the filaments with using different concentrations of the aqueous solution (Scale Bar = 200 μm); (**b**) calculated porosity (%) of the 3D printed filaments; (**c**) Young’s modulus; and (**d**) elongation under 0.01 mol/L, 0.1 mol/L, and 0.5 mol/L Na_2_HPO_4_ conditions. Data expressed as mean ± S.E.M.; * *p*-value < 0.05; ** *p*-value < 0.01; *** *p*-value < 0.005; N.S: non-significant; *n* = 5 for each experimental condition.

Next, we investigated how the HA, formed under the three different aqueous solutions, controlled the structural and mechanical properties of the printed scaffolds. Initially, we performed μCT measurements on the 3D printed filaments, as shown in Figure 4a. Our μCT analysis showed that scaffolds formed in 0.1 mol/L aqueous solutions and 0.5 mol/L aqueous solutions demonstrated about a 1.6-fold decrease in porosity compared to that formed in 0.01 mol/L aqueous solution (Figure 4b), indicating that HA amount formed via different Na_2_HPO_4_ concentrations affected the porosity of the scaffolds. Since the porosity and microstructure of the scaffolds affect the mechanical properties [1,33], we expanded our studies and further characterized the mechanical properties of those scaffolds by performing a 3-points bending test. The 3D printed specimens have been used to obtain the stress-strain curves (Figure S3), and we calculated Young’s modulus and elongation. Our results showed that Young’s modulus value (1162 MPa ± 58 MPa) of the printed specimen soaked in 0.01 mol/L aqueous solution was the lowest, and the specimen soaked in 0.1 mol/L aqueous solutions had the highest Young’s modulus value (1614 MPa ± 66 MPa). However, it was noticeable that Young’s modulus value (1303 MPa ± 107 MPa) of the sample embedded in 0.5 mol/L aqueous solutions was lower than that of one formed in 0.1 mol/L aqueous solutions (Figure 4c).

### 3.4. Osteoclast Differentiation of Cells Embedded in 3D Printed Scaffolds

**Figure 5:**
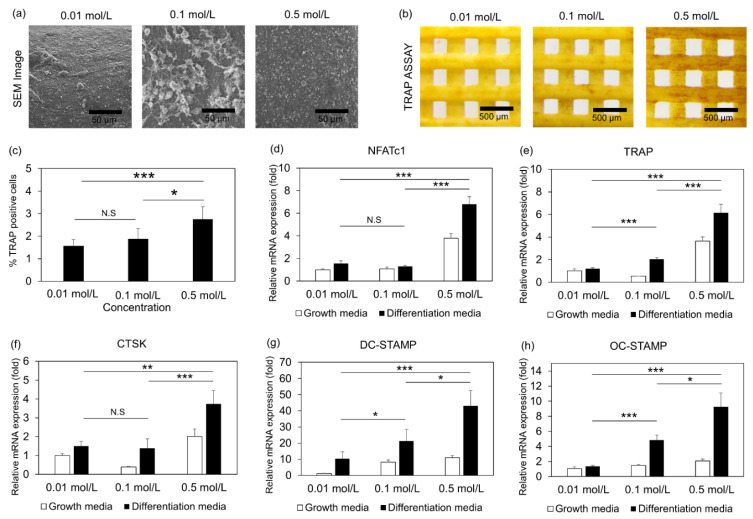
Osteoclastogenesis of 3D printed Scaffolds: (**a**) SEM images of the RAW cells on the scaffold surfaces (Scale Bar = 50 μm); (**b**) Representative images of the Tartrate-Resistant Acid Phosphatase (TRAP) assay (Scale Bar = 500 μm); (**c**) Histogram reports the% TRAP activity; Osteoclast gene expression for (**d**) Nuclear Factor of Activated T Cells 1 (NFATc1); (**e**) TRAP; (**f**) Cathepsin K (CTSK); (**g**) Dendrocyte Expressed Seven Transmembrane Protein (DC-STAMP); (**h**) Osteoclast Stimulatory Transmembrane Protein (OC-STAMP) of the scaffolds embedded in 0.01, 0.1, and 0.5 mol/L Na_2_HPO_4_ solutions. Data expressed as mean ± S.E.M.; * *p*-value < 0.05; ** *p*-value < 0.01; *** *p*-value < 0.005; N.S: non-significant; *n* = 4 for each experimental condition.

**Figure 6:**
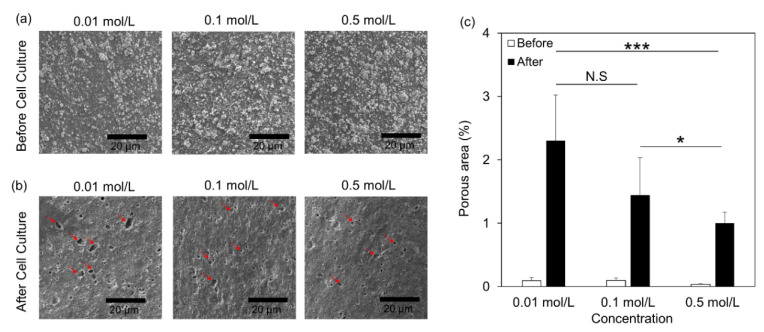
Osteoclast activity in 3D printed scaffolds: SEM images of the formed HA on the scaffold surfaces (**a**) before cell seeding; (**b**) after cell removal (Scale Bar = 20 μm); (**c**) calculation of the porous area on the scaffold surfaces before and after cell seeding. Data expressed as mean ± S.E.M.; * *p*-value < 0.05; *** *p*-value < 0.005; N.S: non-significant; *n* = 10 for each experimental condition.

Finally, we examined the osteoclast activity of those scaffolds by using RAW 264.7 cells which were differentiated into osteoclasts [34,35,36]. SEM imaging demonstrated the cell attachment and spread (Figure 5a), and live/dead assays showed the viability of the cells on those scaffolds after 48 h (Figure S4). Cells lied on scaffolds, prepared at a low concentration (0.01 mol/L aqueous solution), showed lower viability compared to the other conditions (Figure S4). Next, the cells were tested for their osteoclast differentiation potential by TRAP assay (Figure 5b). Interestingly, the cells on scaffolds, prepared in 0.5 mol/L aqueous solutions, showed a 2-fold increase in RAW differentiation potential, indicating that those scaffolds induce osteoclastogenesis (Figure 5c). In addition, we evaluated the gene regulation associated with osteoclast differentiation and activity. To this end, we examined the transcription levels of osteoclast-specific genes, such as Nuclear Factor of Activated T Cells 1 (NFATc1) (Figure 5d), Tartrate-Resistant Acid Phosphatase (TRAP) (Figure 5e), Cathepsin K (CTSK) (Figure 5f), Dendrocyte Expressed Seven Transmembrane Protein (DC-STAMP) (Figure 5g), and Osteoclast Stimulatory Transmembrane Protein (OC-STAMP) (Figure 5h). Specifically, the transcriptional levels of all the gene expressions are significantly higher for the scaffolds generated in 0.5 mol/L solutions than those in 0.01 mol/L solutions (Figure 5). Taken together, our results showed that HA formed under different solutions affected osteoclastogenesis. Finally, after 7d, we examined the resorption of those scaffolds by the differentiated osteoclasts. Specifically, the cells were removed from the surface area of the scaffolds, and SEM images demonstrated the formation of cavities (red arrows) on the surface of those scaffolds (Figure 6a,b). Interestingly, the scaffolds immersed in 0.01 mol/L aqueous solutions demonstrated more and larger voids than other concentrations of the aqueous solutions, and the overall porosity was 2-times higher than 0.5 mol/L aqueous solutions.

Overall, our results demonstrated that tuning HA formation by Na_2_HPO_4_ in 3D printed scaffolds controls microstructure, mechanical properties, and osteoclast activity.

## 4. Discussion

The treatment of bone defects remains a significant challenge, as the options of autografts and allogeneic bone substitutes demonstrate limitations due to high morbidity and low integration with the host tissue, respectively [4,5]. To overcome those limitations, the development of synthetic bone grafts is essential. The use of calcium phosphates (Ca/P), a principal inorganic constituent of natural bone, is a major component of those substitutes. HA and β-TCP, as the most extensively composed Ca/P materials, have been widely tested. However, they fail to meet the clinical requirements of structural support, bone induction, and controllable biodegradability [37,38,39]. For example, HA is osteoconductive but has a slow resorption rate in the physiological environment. Also, β-TCP has a higher resorption rate but is characterized by poor mechanical stability [40,41]. In our study, we overcome those limitations by using novel Ca/P material, which received the US Food and Drug Administration (FDA) approval to be the first commercially available CPC to treat craniofacial defects and bone fractures. This pure apatite without binders or other crystalline phases consists of tightly packed and interlocked nano apatite crystals, has inherently high mechanical strength and demonstrates direct osseointegration at apatite/bone interface with histometrically normal amounts of fibrovascular tissue for the anatomic site [33,42,43,44,45,46,47,48,49]. To this end, in our lab, we have recently established this CPC as a promising source for engineering 3D printed scaffolds [1,50]. Our approach overcomes the requirements of conventional 3D printing methods for high temperature and limited HA composition. The slurry consists of 5 μm TTCP and one μm DCPA particles mixed with PVB/EtOH solution at a high ceramic ratio (75 g/100 mL (%)) and is extruded from 210 μm nozzle into a Na_2_HPO_4_ solution bath and gave us the ability to 3D print high-resolution scaffolds (<210 μm), as described previously [1,50].

Herein, we expand our studies by extruding the CPC/PVB slurry into three different concentrations of Na_2_HPO_4_ solution (0.01 mol/L, 0.1 mol/L, and 0.5 mol/L), resulting in the formation of 3D printed scaffolds with different HA amount through a controllable room-temperature dissolution-precipitation reaction [46]. Previous studies have shown that the CPC precursors, i.e., TTCP and DCPA, are more soluble than HA in neutral pH solutions [46]. In our studies, the three different Na_2_HPO_4_ solution concentrations do not change the pH environment, and the reaction occurs in neutral pH solutions. However, the concentration of Na_2_HPO_4_ solution affected the kinetics of the dissolution-precipitation reaction. Specifically, our data indicate that the HA scaffolds, printed in 0.5 mol/L Na_2_HPO_4_ solutions, formed faster (after three h) than in other conditions. After 12 h, the content of HA is dominant (91.0 ± 2%) in the scaffold with a limited amount of the other residuals. The process of engineering tunable HA scaffolds would impact the way we fabricate grafts, which requires us to fulfill specific criteria for proper bone restoration. To this end, those constructs need to demonstrate pre-defined mechanical properties, pore architecture that may facilitate the exchange of nutrients with the host tissue, and the rate of resorption to be controlled to avoid graft degradation without sufficient new bone formation.

In this study, we identify that the differences in the amount of formed HA correlated with the scaffolds’ porosity and mechanical properties. Although high porosity (i.e., <90%) enhances bone growth and osseointegration of the graft due to large surface area that induces the exchange of bone-inducing factors, it reduces mechanical properties by compromising the structural integrity of the scaffold before new bone formation [51,52,53]. To this end, in our study, we demonstrated that the 3D printed scaffolds have ~40% porosity, indicating the benefit to be applied as grafts. In addition to porosity, the mechanical loading and the degradation rate should be considered parameters for the scaffold’s proper design. Interestingly, our results showed that scaffolds in 0.1 mol/L demonstrated high deformation resistance and less fragile behavior than the other concentrations, indicating that either excessive or reduced HA formation may negatively regulate the mechanical properties of the scaffolds.

Finally, the HA concentration controlled the osteoclast activity. In addition to our previous studies, which showed the osteoconductive potential of those scaffolds [1,50], herein, we demonstrated their osteoclastogenesis and resorption potential. The cells seeded on HA scaffolds, printed in 0.5 mol/L Na_2_HPO_4_ solution, had higher TRAP activity and osteoclast expression, demonstrating a correlation of formed HA with osteoclast activity. Finally, we captured their resorption capacity by evaluating structural changes of the scaffolds developed by the differentiated osteoclasts. Specifically, the scaffolds, printed in 0.5 mol/L Na_2_HPO_4_ solution, showed lower resorption potential indicating that the scaffolds may not be degraded fast and supports the new bone formation [54].

## 5. Conclusions

To conclude, we engineered scaffolds with controlled HA composition using a 3D high-resolution (<210 μm) room-temperature printing methodology. We tuned and dynamically captured the HA formation by controlling the concentration of the Na_2_HPO_4_, which works as an accelerator. The formed HA amounts regulated the scaffold’s mechanical properties, porosity, and osteoclast activity. Overall, this proof-of-concept study further paves the way to produce next-generation bone scaffolds with pre-defined properties which tune bone formation and resorption and enhance graft incorporation in different anatomic locations [1,55].

## 6. Patents

The presented 3D printing method and materials have been submitted to the patent office [US Patent Application number US 2021/0260249 A1] and Patent Cooperation Treaty [(PCT) application number PCT/US2021/019274].

## Data Availability

Not applicable.

## Supplementary Materials

The following supporting information can be downloaded at: https://www.mdpi.com/article/10.3390/jfb13020034/s1, Figure S1: Raman spectra for slurry and 3D printed components; Figure S2: XRD for slurry and 3D printed components; Figure S3: Mechanical analysis of 3D printed scaffolds; Figure S4: Biocompatibility of 3D printed scaffolds. Table S1: 3D printing parameters and apparent viscosity; Table S2: List of primers for RT-qPCR.
