# Supplementary material for: Engineering 3D Printed Scaffolds with Tunable Hydroxyapatite
Source: J Funct Biomater. 2022 Mar 23;13(2):34. doi: 10.3390/jfb13020034 (PMC9036238; doi:10.3390/jfb13020034)
Supplement: Supplementary file 1 [file jfb-13-00034-s001.zip › jfb-1641344-SI.pdf]

Supplementary Materials

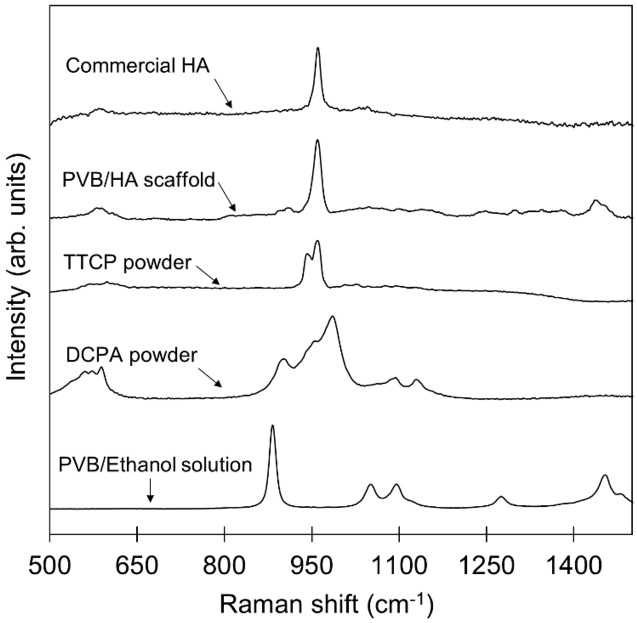

Figure S1. Raman spectra for slurry and 3D printed components. The focused Raman spectra on the fingerprint region of the spectrum between (500 to 1500) cm^{-1} for commercial hydroxyapatite (HA); Poly vinyl butyral (PVB)/HA 3D printed scaffold; Tetracalcium phosphate (TTCP) powder; Dicalcium phosphate anhydrous (DCPA) powder; and PVB/Ethanol (EtOH) solution.

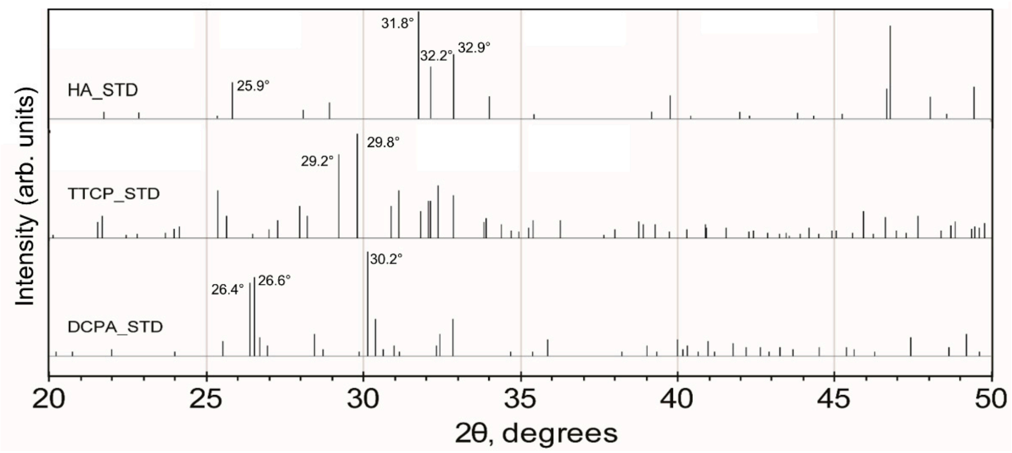

Figure S2. XRD for slurry and 3D printed components. Standard peaks in XRD of HA (blue), TTCP (green), DCPA (red) materials.

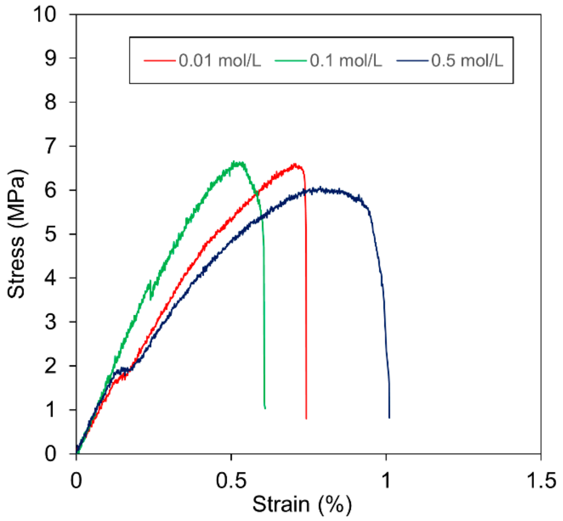

Figure S3. Mechanical analysis of 3D printed scaffolds. Strain-Stress curves of 3D printed scaffolds embedded in 0.01, 0.1 and 0.5 mol/L Na_2HPO_4 solutions.

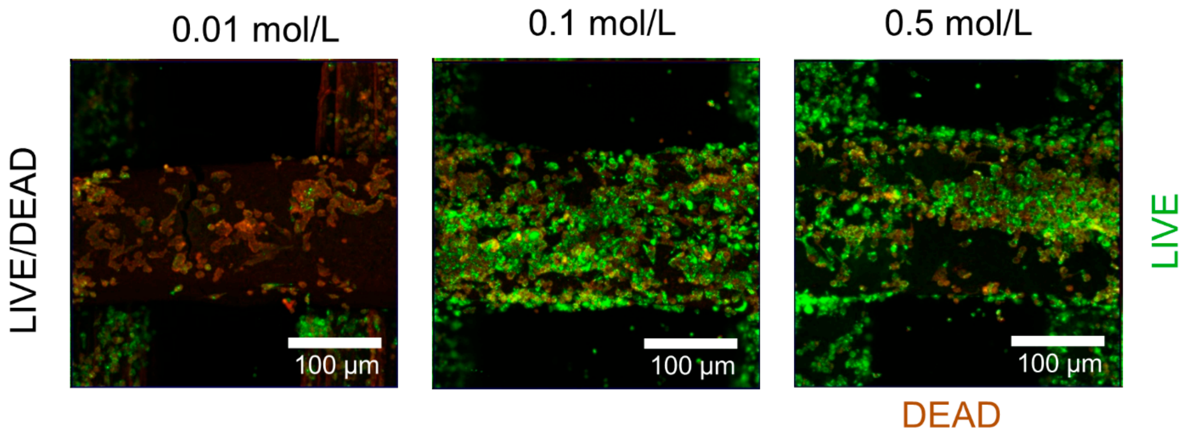

Figure S4. Biocompatibility of 3D printed scaffolds. The RAW cells lied on 3D printed scaffolds embedded in (0.01, 0.1 and 0.5) mol/L Na_2HPO_4 solutions. Live and dead cells were stained for green and red, respectively (Scale Bar = 100 μm).

Table S1. 3D printing parameters and apparent viscosity

	**Values**
Needle diameter (μm)	210
Needle length (mm)	6.35
Printing speed (mm/s)	5
Fill Density (%)	30
Input flow (%)	350
syringe temp (°C)	25
plate temp (°C)	25
Lay-down pattern (°)	0/90
shear rate (1/s)	65.58
viscosity (Pa s)	7.42

Table S2. List of primers for RT-qPCR. NFATC1, Nuclear Factor of Activated T Cells 1; TRAP, Tartrate-Resistant Acid Phosphatase; CTSK, Cathepsin K; OC-STAMP, Osteoclast Stimulatory Transmembrane Protein; DC-STAMP, Dendrocyte Expressed Seven Transmembrane Protein; 18S rRNA, have used in this study.

Gene name		Primer Sequence (5' - 3')	Amplicon Size
human NFATC1	Forward	CACCGCATCACAGGGAAGAC	119
	Reverse	GCACAGTCAATGACGGCTC	
human TRAP	Forward	GACTGTGCAGATCCTGGGTG	122
	Reverse	GGTCAGAGAATACGTCCTCAAAG	
human CTSK	Forward	ACACCCACTGGGAGCTATG	226
	Reverse	GACAGGGGTACTTTGAGTCCA	
human OC-STAMP	Forward	CACCCTGGGTATGGAGCAG	166
	Reverse	CTGGTGAGTGGTATTGAGGAGA	
human DC-STAMP	Forward	CGCTGCCTCCTGGATTATCAC	219
	Reverse	AAGCTCTTTGCCCTTAGGTTG	
human 18S rRNA	Forward	CGGCTACCACATCCAAGGAA	169
	Reverse	GCTGGAATTACCGCGGCT	
